# Capgras and Fregoli syndromes revisited through six different psychiatric clinical cases

**DOI:** 10.1192/j.eurpsy.2023.962

**Published:** 2023-07-19

**Authors:** L. Santa Marinha, P. Felgueiras, A. Miguel, O. Nombora, A. Horta

**Affiliations:** Psychiatry, Centro Hospitalar de Vila Nova de Gaia/Espinho, Vila Nova de Gaia, Portugal

## Abstract

**Introduction:**

Capgras and Fregoli syndromes are delusional misidentification syndromes, characterized by a belief in duplicates and replacements. Capgras delusion was first described by Capgras in 1923, reporting a belief that a person (usually a close relative) has been replaced by an exact double (imposter). On the other hand, Fregoli Syndrome was first described by Courbon and Fail in 1927, and holds a delusion that a familiar person is disguised as a strange person. Several explanatory models have been hypothesized, through myths, psychoanalytical and psychological interpretations, as well as neurobiological explanations.

**Objectives:**

Through six different clinical cases and a narrative review, we aim to revisit the concepts of Capgras and Fregoli syndromes, emphasizing their complexity and heterogenicity.

**Methods:**

We conducted a non-systematic review of recent evidence on Capgras and Fregoli syndromes and expose exemplary clinical cases.

**Results:**

Capgras and Fregoli syndromes are complex psychotic experiences involving a great number of brain areas, with many heterogeneous clinical manifestations and comorbidities. Even though they were initially encompassed in pure psychotic disorders, today they are mainly understood as neurological disorders, in which the delusion primarily results from organic brain lesions or degeneration. Nevertheless, we present several distinct clinical cases with psychiatric diagnoses that include these curious phenomena: a 39-year-old man with schizophrenia; a 67-year-old woman with late-onset schizophrenia; a 24-year-old woman with schizoaffective disorder; a 48-year-old woman with first episode of acute and transient psychotic disorder; a 76-year-old woman with psychotic depression; and a 25-year-old woman with psychosis and intellectual development disorder.

**Conclusions:**

Our review highlights the complexity of the delusional misidentification syndromes. We expose different patients with different psychiatric diagnosis, showing the diversity of pathologies in which these syndromes can fit. Although they seem to be very common in non-psychiatric disorders, little is known about the prognosis and response to treatment or whether there are systematic differences between delusional misidentification syndromes associated with “functional” and “organic” disorders, which should encourage further studies in order to address this gap and provide appropriate care.

**Disclosure of Interest:**

None Declared

